# A utero-pelvic fistula and pelvic-parasitic myoma by hysteroscopic resection of a recurrent submucosal myoma: a case report

**DOI:** 10.3389/fmed.2025.1579105

**Published:** 2025-06-20

**Authors:** YuXi Yan, XiaoRong Chen

**Affiliations:** ^1^Department of Medical Imaging, Jinhua Maternal and Child Health Care Hospital, Jinhua, Zhejiang, China; ^2^Department of Medical Imaging, Jinhua Municipal Central Hospital, Affiliated Jinhua Hospital, Zhejiang University School of Medicine, Jinhua, Zhejiang, China

**Keywords:** fibroid uterus, female, hysteroscopic, treatment, myomectomy

## Abstract

**Background:**

The occurrence of a uterine fistula combined with parasitic myoma formation is rare. We report a case involving a utero-pelvic fistula and a pelvic-parasitic myoma following hysteroscopic resection of a recurrent submucosal myoma.

**Case presentation:**

A 37-year-old woman who had undergone surgery for laparoscopic myomectomy of large uterine fibroids presented with abnormal uterine bleeding (AUB) for over 6 months and 4 years ago. One year ago, the patient returned to the hospital due to abnormal uterine bleeding (AUB); both transabdominal sonography (TS) and dynamic pelvic magnetic resonance imaging (MRI) revealed submucosal myomas that were subsequently removed hysteroscopically. On a subsequent visit, the patient returned for the third time due to recurrent AUB. She eventually underwent a laparoscopic myomectomy of uterine fibroids after transabdominal sonography (TS) detected a uterine myoma, and dynamic pelvic MRI revealed a uterine myoma at the base of the uterus that locally penetrated the uterine serosal surface, as well as a small leiomyoma in the left utero-rectal fossa.

**Conclusion:**

In rare cases, recurring submucosal myoma after hysteroscopic surgery can breach the surface of the uterine serosa, leading to utero-pelvic fistula and pelvic-parasitic myoma. Preoperative enhanced MRI examinations can aid clinicians in choosing appropriate surgical methods, thereby avoiding the risk of misdiagnosis and missed diagnosis.

## 1 Introduction

Uterine myomas are the most common benign pelvic tumors in women and represent the most common indication for both laparoscopic and hysteroscopic surgery. Despite the effectiveness of surgery, the rate of recurrence of uterine fibroids following laparoscopic myomectomy has been reported to be as high as 52.9% according to one publication, and these fibroids often require re-surgical intervention in women experiencing recurrent uterine myomas (1).

Hysteroscopic myomectomy is the most preferred approach for excising submucosal myomas due to its efficacy, safety, and low risk of surgical complications (2). Preoperative assessment of the fibroid’s relationship to the uterine endometrium, the extent of penetration into the uterine myometrium, and the pedunculated character of the uterine myomas are necessary for cancer stages FIGO 1 and FIGO 2 in order to make surgery safer (2).

According to the recent literature reports, there are two ways in which parasitic myomas occur: first, when laparoscopic morcellation can seed leftover tissue fragments into the abdominal cavity, they are then often found in either the abdominal cavity or port site. The overall incidence of iatrogenic parasitic myomas ranges from 0.12 to 0.95% (3). The second type of spontaneous parasitic leiomyomas is less common, and the pedunculated subserosal myomas sporadically separate from the uterus and then adhere to any area of the pelvic blood supply (4).

## 2 Case presentation

**Figure 1:**
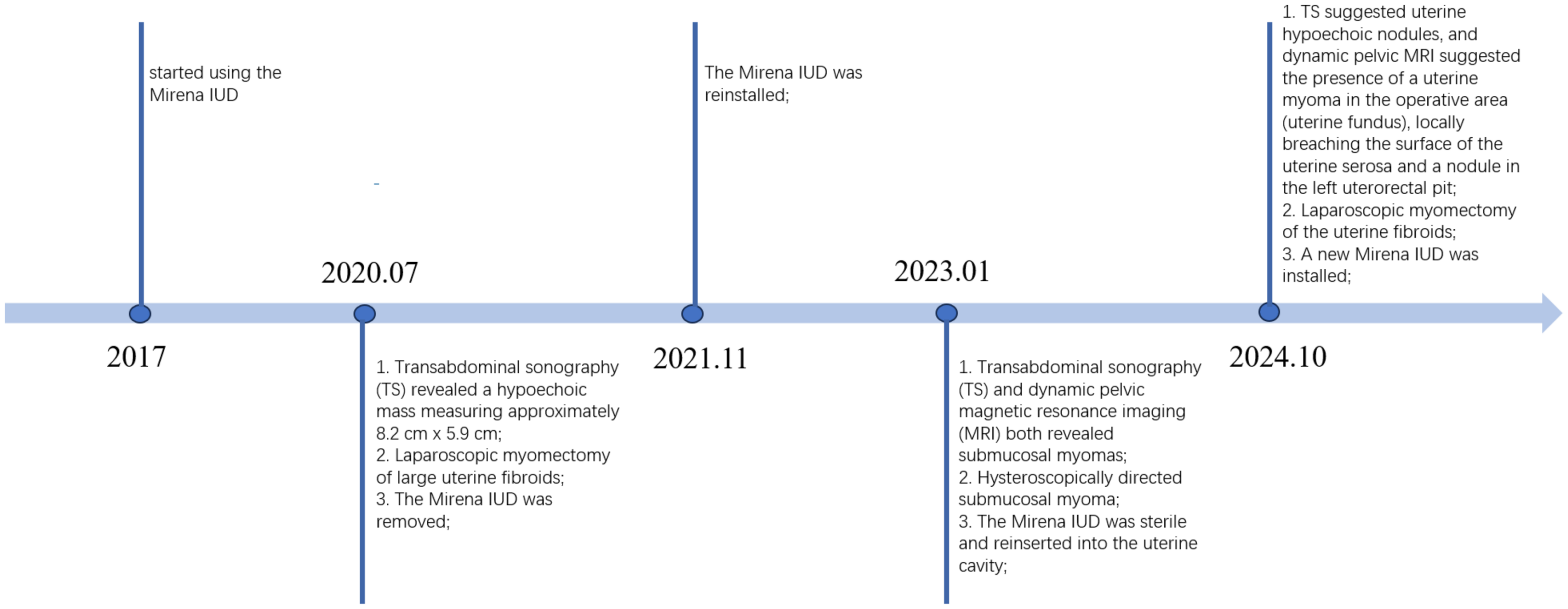
Chronological timeline of clinical events.

In July 2020, a 37-year-old woman of childbearing age consulted for AUB of more than 6 months, with an irregular menstruation (a menstrual cycle of 20 days and menses duration of 10–12 days), a moderate amount of menstrual flow, and no dysmenorrhea. The woman had no prior medical or surgical history and delivered two children vaginally in 2012 and 2014. The woman usually felt dizzy and fatigue, with an Hb of 77 g/L (i.e., moderate anemia). Upon gynecological examination, her uterus was positioned anteriorly and enlarged to emulate a size consistent with a uterus at over 2 months of pregnancy. We observed a hypoechoic mass measuring approximately 8.2 cm × 5.9 cm in the right intramural uterus by TS. The patient’s large uterine fibroid, together with clinical signs (abnormal uterine bleeding and moderate anemia), indicated the need for a laparoscopic myomectomy to remove the fibroid. During surgery, a smooth nodule suggestive of a fibroid tumor was observed penetrating from the submucosa of the uterine anterior wall. It was 8 cm x 6 cm in size and did not adhere to the surrounding tissues. During uterine fibroid resection, an incision was made in the front wall of the uterus, and a tumor cavity was discovered that invaded deeply into the uterus; the uterine fibroid was subsequently removed by morcellation after the suture closure of the tumor cavity. Pathological results confirmed uterine leiomyoma. In 2017, the patient started using the Mirena intrauterine device IUD for family planning. The Mirena IUD was reinstalled in November 2021 after being removed during laparoscopy in 2020 (Figure 1).

**Figure 2:**
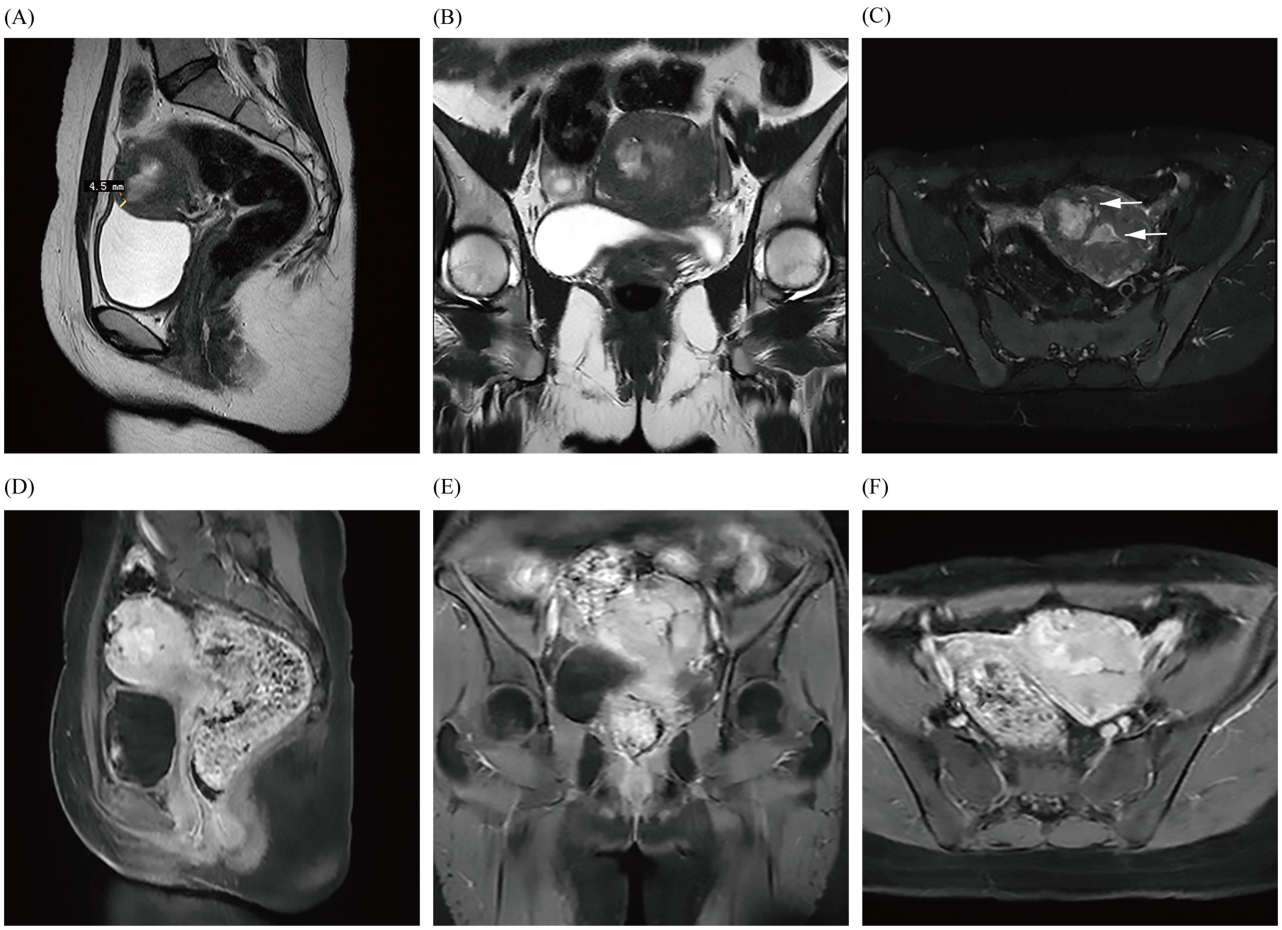
Images **(A–F)** show the MRI in January 2023, demonstrating a fibroid with just under a 50% submucosal component (arrows) that is closest to the uterine serosa layer by approximately 4.5 mm; they also reveal obvious enhancement of the fibroid.

In January 2023, the patient was admitted once again for AUB that had persisted for 10 days and reported experiencing heavy menstrual flow that was dark reddish in color and dysmenorrhea during menses. Laboratory tests showed Hb to be within normal limits, and gynecological examination revealed no notable abnormalities. TS and dynamic pelvic MRI (Figure 2) were suggestive of a submucosal myoma. The patient’s fibroid-related symptoms (AUB) reappeared due to the recurrence of the submucosal myoma, which indicated hysteroscopically directed submucosal myoma. During subdivided electrocautery, fibroid tumor-like tissue was identified in the left anterior wall and fundus of the uterine cavity, but no evidence of uterine rupture was observed. The resected uterine fibroid was approximately 2.5 cm x 2.0 cm in size, and approximately 5% of it was convex to the uterine cavity, with cystic degeneration. The pathological results confirmed submucosal myoma.

**Figure 3:**
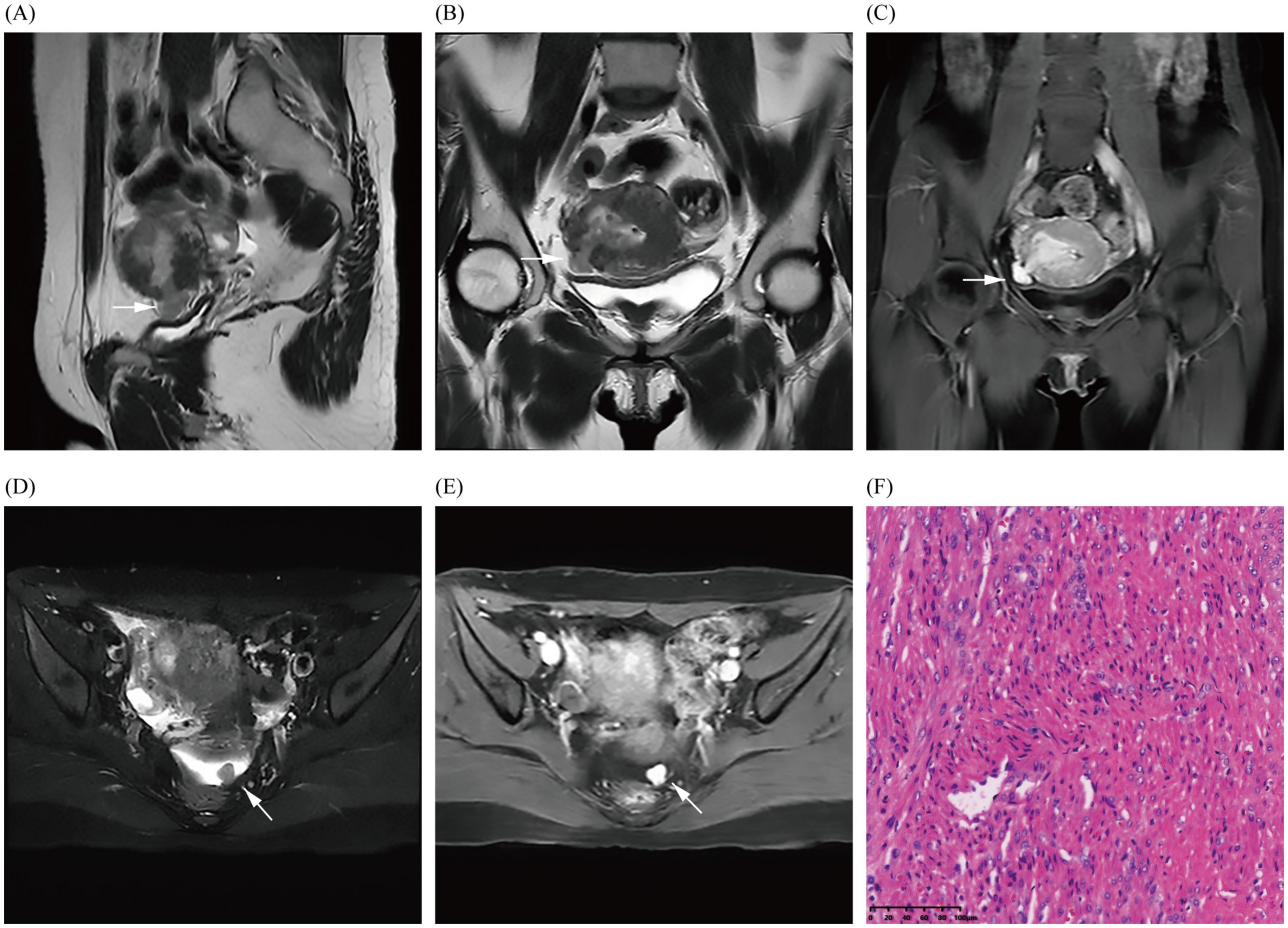
Images **(A–E)** show the MRI in October 2024 and demonstrate the fibroid breaking through the uterine serosa layer (indicated by arrows); a nodular abnormal signal shadow is also observed in the left rectouterine fossa, revealing obvious enhancement of the fibroid. Image **(F)** shows the pathological results (uterine and pelvic) confirming uterine leiomyoma through hematoxylin and eosin staining of the fibroid (magnification × 200).

In October 2024, the patient presented with AUB for the third time, but without dysmenorrhea, and with a heavy menstrual flow that lasted for 1.5 months and was bright red in color and moderate in volume. Laboratory tests and gynecological examination yielded normal results. TS suggested uterine hypoechoic nodules, and dynamic pelvic MRI (Figure 3) suggested the presence of a uterine myoma in the operative area (uterine fundus), locally breaching the surface of the uterine serosa and spanning the uterine cavity, myometrium, and pelvis. MRI also revealed a nodule in the left uterorectal pit, which was *de novo* in nature (which was in contrast to that observed under previous imaging), and based on its imaging characteristics, it was presumed to be a fibroid. Imaging revealed the presence of a utero-pelvic fistula and a nodule in the left-rectouterine fossa, which facilitated the clinician’s choice of surgical method (i.e., laparoscopic myomectomy of the uterine fibroids). During intraoperative laparoscopic exploration, the uterine appeared enlarged, and the uterine cavity revealed fibroid tumor-like tissue growth that extended to the right broad ligament; and only the uterine fibroid-like tissue identified by MRI was ultimately uncovered. The pathological results (uterine and pelvic) confirmed leiomyoma. At 5-month postoperative follow-up, the patient’s preoperative symptoms had completely resolved, and she was experiencing regular, tiny, reddish menstrual cycles without dysmenorrhea. Additionally, both transabdominal sonographies indicated no significant uterine or adnexal abnormalities.

## 3 Discussion

The development of uterine fistulae is typically associated with factors such as trauma or infection, and some patients with a history of cesarean section are at risk of uterine rupture or uteroperitoneal fistula due to severe scarring of the myometrium (5). Additionally, there is a possibility of the occurrence of uterocolonic fistula in patients with congenital uterine malformations in the region in which the IUD is inserted (6).

In our case, the young patient had no history of cesarean section or congenital malformations of the uterus, used a Mirena IUD within its expiration date, and showed a history of hysterectomy for uterine fibroids within three generations of her mother. In 2001, De Iaco et al. reported a case in which transcatheter embolization of uterine arteries caused localized tissue necrosis and ischemic contraction of the uterine myometrium postoperatively, and they observed a full-thickness discontinuity of the uterine wall in the location of previous hysteroscopically directed submucosal myomas, as detected through hysteroscopy (7). In one case, the patient’s pelvic MRI enhancement before hysteroscopy suggested localized thinning of the anterior wall of the uterus, with the thinnest area measuring at only 4.5 mm. However, the enhancement of ≥80% of the scar area indicated that the blood flow in the scar area of the uterine myometrium was equivalent to the flow in normal tissues (5). The potential mechanism for the uteropelvic fistulae found in the patient was also similar to the mechanism underlying fistula formation after uterine artery embolization. In January 2023, dynamic pelvic MRI revealed that the submucosal myoma was located approximately 4.5 mm from the uterine serosa. During the subsequent hysteroscopic myomectomy, the excised leiomyoma was found to penetrate deeply into the surface of the uterine serosa, and the heat of the electrocautery of the uterine leiomyomas damaged or decreased the elasticity of the muscle wall; these conditions may have led to a weakened area, posing a risk factor for the formation of uterine fistulae. Subsequently, recurrent submucosal myomas can easily grow through this weakened area into the pelvic cavity. The patient’s symptoms were not severe, likely due to the lack of menstrual blood flow into the pelvic cavity.

In addition, at her recent preoperative pelvic MRI, the patient was discovered to have a small fibroid in the left rectouterine fossa that had not been detected at the time of her hysteroscopy in January 2023, approximately 3 years or more after the first laparoscopic myomectomy. When we systematically reviewed the literature, we discerned that the median size of iatrogenic parasitic myomas diagnosed after laparoscopic myomectomy was 20–50 mm, with a median time between surgery and diagnosis of 48 months; and that the myomas were often located in the abdominal cavity or port site (3). If the fibroid had been an iatrogenic parasitic myoma resulting from residual tissue debris following the first laparoscopic myomectomy, then this myoma should have been detected on the preoperative MRI performed before hysteroscopy in 2023. However, it was not detected, and we therefore propose another possibility. It is reasonable to assume that this represents a particular type of spontaneous parasitic leiomyomas. In our case, there were two differences from spontaneous parasitic leiomyomas: first, the patient had a history of laparoscopy-related surgery; and second, the main body of the fibroid was in the uterine cavity and myometrium, with a very small percentage of extra-serosal and intra-serosal localization. However, the typical growth pattern of spontaneous parasitic leiomyoma was similar to that found in our case (2). The patient’s recurrent submucosal myomas extended outward from the uterine cavity, eventually penetrating the uterine serosal layer, and portions of the myomal tissues were exposed within the pelvic cavity; some of these myomatous tissues may have lost their blood supply from the uterus and detached, subsequently only surviving via a blood supply from the peritoneum on the left side of the pelvis and eventually developing parasitic leiomyomas. To the best of our knowledge, no imaging-based literature has reported a case of a patient undergoing laparoscopic and hysteroscopic myomectomy with a recurrent submucosal fibroid that communicated with the uterine and pelvic cavities, resulting in the formation of a utero-pelvic fistula and a parasitic myoma. Furthermore, these myomas should also be differentiated from diffuse uterine leiomyomatosis (3).

The patient’s TS only suggested uterine fibroids, and we did not observe the formation of a utero-pelvic fistula, whereas MRI clearly showed the disruption of the continuity of the uterine serosa. Color Doppler hysterosonography can be used in patients with clinical suspicion of uterine fistula to observe and help clarify the presence or absence of fistula formation (8). Although it is possible to visualize uterine fistulas by injecting saline into the uterine cavity, the small ultrasonographic field of view, the restricted sound window, and the interference of gas in the intestinal lumen affect the diagnostic assessment of uterine fistulas and do not allow for the visualization of parasitic myoma. In addition, saline was not injected in routine pelvic ultrasonography. Ultrasonography also has its limitations. However, MRI images stored in Picture Archiving and Communication Systems can be accessed anytime and from anywhere, thereby facilitating communication and diagnosis between radiologists and clinicians. Moreover, pelvic MRI is valuable for postoperative follow-up, enabling the evaluation of the degree of incision healing and the location and size of recurrent fibroids. In summary, MRI can be used as a non-invasive method to clearly show uterine fistulas in multiple planes and directions (9). It also exhibits the ability to assess the peripelvic area and degree of healing of the uterine incision postoperatively, and it can assist in the clinical management of uterine fistulas by facilitating early intervention.

## 4 Conclusion

We posit that examination with MRI reflects obvious advantages in improving the preoperative detection rate of uterine fistulae, and allows the evaluation of the peripelvic situation. MRI also facilitates the diagnosis of uterine fibroids and allows for the classification and evaluation of postoperative uterine incision recovery, thereby aiding clinical decision-making to select appropriate treatment methods and prevent uterine rupture or the formation of uterine fistulae.

## Data Availability

The original contributions presented in the study are included in the article/supplementary material, further inquiries can be directed to the corresponding author.
