# Correction: A utero-pelvic fistula and pelvic-parasitic myoma by hysteroscopic resection of a recurrent submucosal myoma: a case report

**DOI:** 10.3389/fmed.2025.1688770

**Published:** 2025-09-01

**Authors:** YuXi Yan, XiaoRong Chen

**Affiliations:** ^1^Department of Medical Imaging, Jinhua Maternal and Child Health Care Hospital, Jinhua, Zhejiang, China; ^2^Department of Medical Imaging, Jinhua Municipal Central Hospital, Affiliated Jinhua Hospital, Zhejiang University School of Medicine, Jinhua, Zhejiang, China

**Keywords:** fibroid uterus, female, hysteroscopic, treatment, myomectomy

Affiliations 1 “Department of Medical Imaging, Jinhua Maternal and Child Health Care Hospital, Jinhua, Zhejiang, China”, and 2 “Department of Medical Imaging, Jinhua Municipal Central Hospital, Affiliated Jinhua Hospital, Zhejiang University School of Medicine, Jinhua, Zhejiang, China” were erroneously given as 1 “Department of Radiology, Jinhua Maternal and Child Health Hospital, Jinhua, Zhejiang, China”, and 2 “Department of Radiology, Jinhua Central Hospital, Zhejiang University Affiliated Jinhua Hospital, Jinhua, Zhejiang, China”.

The original version of this article has been updated.

